# Invasive pulmonary aspergillosis 10 years post bone marrow transplantation: a case report

**DOI:** 10.1186/1752-1947-3-26

**Published:** 2009-01-26

**Authors:** Rifat Rashid, David W Denning

**Affiliations:** 1 Faculty of Medicine and Human Sciences, University of Manchester, Wythenshawe Hospital, Manchester, UK; 2 School of Medicine, Education and Research Centre, the University of Manchester, Manchester, UK

## Abstract

**Introduction:**

Invasive pulmonary aspergillosis is a leading cause of mortality and morbidity in bone marrow transplant recipients. Establishing the diagnosis remains a challenge for clinicians working in acute care setting. However, prompt diagnosis and treatment can lead to favourable outcomes

**Case presentation:**

We report a case of invasive aspergillosis occurring in a 39-year-old Caucasian female 10 years after an allogeneic haematopoietic bone marrow transplant, and 5 years after stopping all immunosuppression. Possible risk factors include bronchiolitis obliterans and exposure to building dust (for example, handling her husband's dusty overalls). There are no similar case reports in the literature at this time.

**Conclusion:**

High clinical suspicion, especially in the setting of failure to respond to broad-spectrum antibiotics, should alert clinicians to the possibility of invasive pulmonary aspergillosis, which, in this case, responded to antifungal therapy.

## Introduction

Clear cut risk factors for developing invasive pulmonary aspergillosis (IPA) include neutropenia, immunosuppressive therapy, cytomegalovirus infection and chronic graft versus host disease. IPA in patients after allogeneic haematopoietic stem cell transplant (HSCT) is associated with a poor prognosis and diagnosis is often delayed. Mortality rates have been reported to be in the region of 87 to 90% [1-4].

Pulmonary complications of post-HSCT are generally divided into two groups: early (<100 days) and late (>100 days) complications. The "high-risk" period is described as being 6 to12 months after bone marrow transplant (BMT) with resolution of immune impairment 12 to15 months post-allogeneic HSCT [5].

Median time to diagnosis is generally thought to be in the region of 60 to100 days after allogeneic transplantation. Although cases are described infrequently in the literature of IPA beyond 100 days, rarely has the time exceeded 210 days. One case occurring 470 days after HSCT of *Aspergillus ustus *infection has been reported [6]. However, presentation 10 years post-HSCT and 5 years post cessation of immunosuppression is exceptionally rare. Numerous prospective and retrospective studies of pulmonary complications following HSCT do not include a similar case.

## Case presentation

We report the case of a 25-year-old Caucasian lady who underwent a matched related donor stem cell transplant in June 1994 for underlying chronic myeloid leukaemia in the first chronic phase. She was conditioned with cyclophosphamide total body irradiation and received recombinant DNA- monoclonal antibody (Cam-Path1H). She received fungal prophylaxis with fluconazole at a dosage of 100 mg per day for 6 months post transplant. In January 1996 she experienced cytogenetic relapse with Philadelphia positivity and subsequently received four doses of donor lymphocyte infusions. In mid-1997, she started to complain of poor exercise tolerance and lung function tests revealed FEV_1 _1.05 litres (predicted 3.28, % predicted = 32) VC 2.2, (predicted 3.78, % predicted = 58) RV 2.4 and DLCO 5.8 (predicted 8.1, % predicted 72) interpreted as moderate airflow obstruction according to GOLD criteria. An open lung biopsy revealed evidence of obliterative bronchiolitis thought to be a consequence of the donor lymphocyte infusions received previously. Treatment with cyclosporin and corticosteroids was commenced. Her lung function improved (DLCO improved to 6.2); however, she developed marked steroid-induced myopathy.

In April 1998, her condition deteriorated with frequent chest infections and worsening lung function (DLCO 5.76). This required a further increase in her steroid dose. In July 1998 she required intravenous immunoglobulin infusions for hypogammaglobulinaemia. The frequency of chest infections decreased gradually in response to this with a concomitant symptomatic improvement in her lung function and exercise tolerance. By autumn 1999 her steroid therapy was being tapered off.

Unfortunately, she did not enjoy good health for long; in 2001 she suffered recurrent bouts of bronchitis and occasional pneumonia. Her lung function tests were consistent with airflow obstruction caused by bronchiolitis obliterans thought to be a sequel to graft versus host disease (GVHD).

A computed tomography (CT) scan of her thorax in May 2001 confirmed changes consistent with the above and also a number of pulmonary nodules in the left upper lobe and evidence of bronchiectasis. At this point her immunoglobulin levels were noted to be within normal limits and she did not require replacement. Immunosuppressant therapy was discontinued in 1999–2000.

In September 2005 at the age of 39, she was admitted to our hospital with shortness of breath, wheeze, pyrexia, cough productive of yellow sputum and pleuritic chest pain. Her arterial oxygen saturations were 95% on room air. Her chest radiograph showed right mid-zone and basal opacification and loss of costophrenic angle. Although her total white blood cell count was normal, her C-reactive protein was raised at 269 (normal <10 mg). Fever continued despite broad-spectrum antibiotic therapy with intravenous third generation cephalosporin, piperacillin and macrolide therapy.

Her CD4 count was normal with a normal lymphocyte profile. No antibodies were detected to common causes of atypical and viral pneumonia. Absolute neutrophil count (ANC) and white blood cell (WBC) on admission were 18,280 mm^3 ^and 25,400 mm^3 ^respectively with lymphocyte count of 3160 mm^3^. CD4 cell count was also within normal limits. Immunoglobulin levels revealed a high globulin level 45 g/L, IgG 20.1 g/L, IgM 4.4 g/L all within normal limits.

Sputum cultures grew *Aspergillus fumigatus *in three samples which was fully susceptible to itraconazole, voriconazole and amphotericin B.

**Figure 1:**
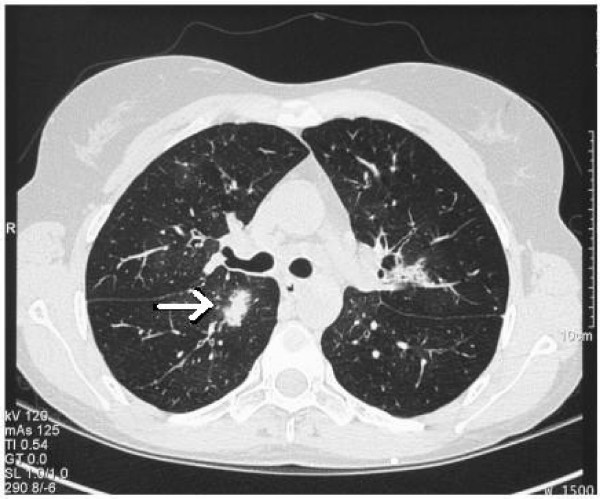
**September 2005(CT Thorax): Halo sign**. Note the so-called "halo sign", seen as a blush around the lesion. This sign is indicative of haemorrhage and highly suggestive of infection with an angioinvasive fungal organism.

**Figure 2:**
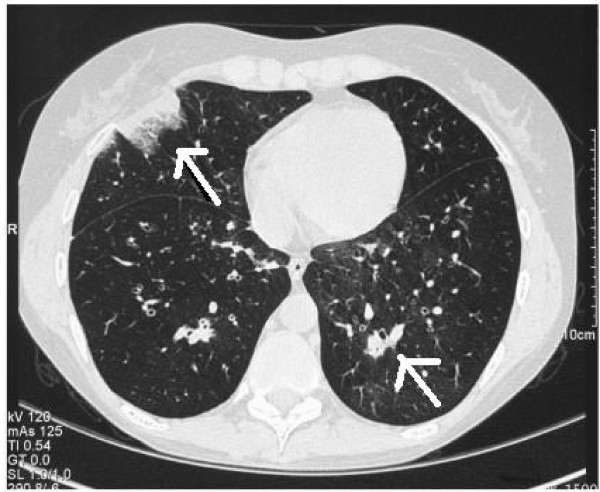
**September 2005 (CT Thorax): Multifocal areas of infection**.

CT scan of the thorax showed multifocal air space shadowing with at least 20 areas of disease and right upper lobe consolidation. In addition, classical findings of halo sign and signet ring sign were reported (Figures 1 and 2), in conjunction with strongly positive serology results (Table 1). Her initial results included negative serum galactomannan and negative blood Aspergillus PCR test but strongly positive precipitins to *A. fumigatus *at a dilution level of 1/256.

**Table 1:** *Aspergillus *precipitins test results over time.

Date	Aspf 2109	Aspf 2140	Dilution
Sep 2005	detected	detected	1/256
Dec 2005	detected	detected	1/64

The diagnosis of invasive aspergillosis was made. She was treated with intravenous voriconazole only.

**Figure 3:**
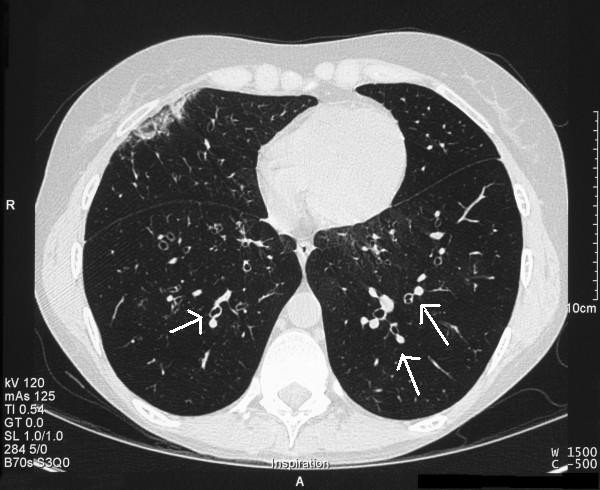
**October 2005 (CT Thorax): Resolution of areas of consolidation and "signet ring sign"**. The signet ring sign is a finding seen on CT scans of the thorax. It consists of a small circle of soft tissue attenuation that abuts a ring of soft tissue attenuation surrounding a larger low attenuating circle of air and is indicative of bronchiectasis.

Follow-up CT scans in Oct 2005 (Figure 3) and more recently February 2006 showed marked improvement in the original areas of invasive aspergillosis and some residual ground-glass shadowing. She continues on oral voriconazole therapy and has made remarkable clinical progress. This is also reflected in her immunological and radiological markers (Table 1).

Subsequent to her discharge, her husband raised the possibility of major exposure to dust following her handling his overalls prior to her illness. He had been involved in partial house demolition and his clothes were extremely dusty. She received 9 months of voriconazole with a trough concentration of 1,900 ng/mL and post-dose concentrations varying from 2,030 to 3,860 ng/mL. Her *Aspergillus *precipitin titre fell gradually to 1/2 at the end of therapy, and was unchanged a year after discontinuation of therapy.

## Discussion

Pulmonary infection or chronic graft versus host disease leading to bronchiolitis obliterans as in our case could predispose to IPA. It is certainly recognised as a late non-infectious complication known to occur in 2 to 10 % of bone marrow transplant recipients which occurs almost exclusively in allogeneic BMT or stem cell recipients. IPA 10 years post HSCT is exceptionally rare regardless of aetiology.

Signs and symptoms may be confused with routine chest infections and include: dyspnoea, initially on exertion and later at rest, cough, wheeze and fever. Chest X-ray findings include hyperinflation. High-resolution CT of thorax is more informative. Typical features include focal or diffuse areas of decreased parenchymal attenuation (mosaic attenuation), bronchial wall thickening and air trapping.

Pulmonary function tests show obstructive patterns with low FEV1 and low FEV1/FVC ratio. In addition low diffusion capacity is noted. Most clinicians base the diagnosis on the aforementioned CT and pulmonary function findings in the setting of allogeneic BMT. Again, survival rates are not favourable in this cohort of patients.

Major exposure to *Aspergillus *spores may contribute to the risk. Contaminated air-ventilation systems have been implicated in clusters of infection within departments [7]. Hospital water could be a major source of filamentous fungi, in particular *Aspergillus fumigatus *[8-10]. We have recently noted 'primary' *A. fumigatus *infection after exposure to bark chippings presumably heavily contaminated with fungus in normal people [11].

The diagnosis of IPA always remains a challenge in immunocompromised patients, often only made post-mortem. In our case we feel the diagnosis of IPA is merited as defined by the recently published European Organization for Research and Treatment of Cancer/Mycoses Study Group (EORTC-MSG) criteria. The revised guidelines suggest that a diagnosis of probable invasive fungal disease can be made on the basis of host factors, clinical features and mycology [12].

In this case our patient had received allogeneic HSCT, had chronic GVHD, and had had prior treatment with T-cell immunosuppressant drugs (Cam-Path 1H). However, she was not deemed to be immunocompromised at the time of diagnosis. She did present with a pyrexial illness unresponsive to broad spectrum antibiotic therapy. As regards to clinical features, she had clinical signs and radiological signs consistent with lower respiratory tract infection. CT findings confirmed the presence of halo signs and multi-focal areas of air space shadowing and evidence of bronchiolitis obliterans. Mycological samples in the form of three separate sputum cultures revealed *Aspergillus fumigatus *which was fully sensitive. In addition the strongly positive *Aspergillus *precipitins to *A. fumigatus*, makes it likely that this patient had probable invasive aspergillosis on the basis of EORTC-MSG criteria (despite not being immunocompromised) although on clinical grounds there was no doubt that the diagnosis was one of IPA. Other features that should alert physicians to the possibility of IPA include sinus infection, ear pain or discharge, facial pain or swelling, localised pallor of nasal septum or turbinate mucosa, and orbital symptoms. *Aspergillus *may invade pulmonary vasculature leading to haemoptysis or pulmonary haemorrhage which may be the presenting complaint.

The role of *Aspergillus *precipitins and polymerase chain reaction (PCR) in the diagnosis of IPA remains undefined.

## Conclusion

Radiological, serological and respiratory samples helped in making the diagnosis of IPA in this case and were instrumental in the commencement of appropriate anti-fungal therapy. Often treatment has to be started on the basis of clinical suspicion in order to prevent poor outcome. Our case highlights that even after 10 years post HSCT, the diagnosis of IPA should be considered and is treatable.

## Abbreviations

CT: computed tomography; IPA: invasive pulmonary aspergillosis (IPA); HSCT: haematopoietic stem cell transplant; BMT: bone marrow transplant; ANC: absolute neutrophil count; WBC: white blood cell; PCR: polymerase chain reaction; DLCO: diffusing capacity of the lung for carbon monoxide; GVHD: graft versus host disease; EORTC-MSG: European Organization for Research and Treatment of Cancer/Mycoses Study Group.

## Consent

Written informed consent was obtained from the patient for publication of this case report and accompanying images. A copy of the written consent is available for review by the Editor-in-Chief of this journal.

## Competing interests

The authors declare that they have no competing interests.
